# A Kinetic Isotope Effect in the Formation of Lanthanide Phosphate Nanocrystals

**DOI:** 10.1021/jacs.2c02424

**Published:** 2022-05-20

**Authors:** Gal Schwartz, Uri Hananel, Liat Avram, Amir Goldbourt, Gil Markovich

**Affiliations:** † School of Chemistry, Raymond and Beverly Sackler Faculty of Exact Sciences, Tel Aviv University, Tel Aviv 6997801, Israel; ‡ Department of Chemical Research Support, Weizmann Institute of Science, Rehovot 7610001, Israel

## Abstract

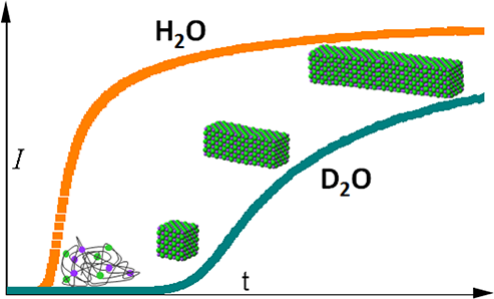

Mechanisms of nucleation and growth of crystals are still attracting a great deal of interest, in particular with recent advances in experimental techniques aimed at studying such phenomena. Studies of kinetic isotope effects in various reactions have been useful for elucidating reaction mechanisms, and it is believed that the same may apply for crystal formation kinetics. In this work, we present a kinetic study of the formation of europium-doped terbium phosphate nanocrystals under acidic conditions, including a strong H/D isotope effect. The nanocrystal growth process could be quantitatively followed through monitoring of the europium luminescence intensity. Hence, such lanthanide-based nanocrystals may serve as unique model systems for studying crystal nucleation and growth mechanisms. By combining the luminescence and NMR kinetics data, we conclude that the observed delayed nucleation occurs due to initial formation of pre-nucleation clusters or polymers of the lanthanide and phosphate ions, which undergo a phase transformation to crystal nuclei and further grow by cluster attachment. A scaling behavior observed on comparison of the H_2_O and D_2_O-based pre-nucleation and nanocrystal growth kinetics led us to conclude that both pre-nucleation and nanocrystal growth processes are of similar chemical nature.

## Introduction

The kinetic isotope effect (KIE) is a phenomenon associated with the sensitivity of reaction rates to isotopic substitution of reactants (or solvents). Formally, it is defined as the ratio of the reaction’s rate constants, i.e., *k*_L_/*k*_H_ where L and H correspond to the light and heavy isotopes, respectively. KIE has been studied for decades as a powerful tool for clarifying reaction mechanisms, particularly when studying the properties of the transition states.^1,2^ Since a strong isotope effect is associated with forming or breaking of bonds involving the isotopically substituted atoms, it informs about which bond is formed or broken, elucidating the reaction pathway.^3−5^ Previous works regarding biochemical processes isolated from organisms for in vitro laboratory studies have shown pronounced changes in the reaction rate upon changing the solvent from H_2_O to D_2_O.^6^ Similar effects were also studied for hydrolysis reactions in organometallic systems and for the enzymatic oxidation of alcohols and amines.^7^ The heart of solvent-induced KIE lies in the zero-point energy difference between the two molecules. The net difference in the activation energies between isotopically substituted reactants should reflect the sum of all bonding changes.^6^

Inorganic nanocrystals (NCs) often serve as model systems for studies of crystallization and molecule–crystal surface interactions. However, the initial steps of nucleation and growth of colloidal NCs are hard to monitor due to the very small size of atomic clusters that form the crystal nuclei and their complex dynamics in solution. While macroscopically grown crystals are generally considered thermodynamically stable, the processes governing the initial nuclei formation often occur under supersaturation conditions and are therefore kinetically controlled.^8,9^ As nucleus sizes are typically in the (sub-)nanometer regime, the surface energy plays an important part in their formation or dissociation.

There are several models describing crystal nucleation and growth. In La Mer’s model, nucleation and growth are separated into two stages.^10,11^ It is described as an initial increase in free monomer/precursor concentration up to a critical level where a burst of crystal nucleation occurs, quickly lowering the precursor’s concentration below the critical level, then allowing for a relatively slow growth of the formed nuclei. On the other side, there are continuous nucleation models, such as the Finke–Watzky model,^12^ which is characterized by two pseudo-elementary steps corresponding to slow continuous nucleation from precursor A (A → B, rate constant *k*_1_) in parallel to autocatalytic growth of B nuclei and formation of B particles (A + B → 2B, rate constant *k*_2_).

It is conceivable that monitoring the KIE in the early stages of NC formation could reveal new mechanistic aspects about these processes. While solvation properties are extremely important for crystal growth, only two studies have previously reported the solvent isotope effect in gold nanocrystal formation.^13,14^ Here, we present an H/D KIE in the formation of inorganic salt nanocrystals under acidic conditions. The present study followed the growth kinetics of Eu^3+^-doped TbPO_4_·H_2_O colloidal nanocrystals in real time using photoluminescence spectroscopy. We found that exchanging H_2_O for D_2_O as the solvent, and probably more important, exchanging H_3_PO_4_ for D_3_PO_4_ as the reactant had strongly affected the nanocrystal nucleation and growth rates.

## Experimental Section

The synthetic procedure was previously described by Hananel *et al.*(15) Briefly, 1742 μL of 28 mM solution of TbCl_3_·6H_2_O and EuCl_3_·6H_2_O (Tb:Eu molar ratio of 95:5%) in H_2_O or D_2_O or their mixture was heated to the desired reaction temperature, after which 292 or 146 μL of 34 mM Na_2_HPO_4_ solution (corresponding to 2:1 or 1:1 phosphate:lanthanide mole ratio, respectively), heated to the synthesis temperature, was rapidly added to the lanthanide solution while stirring. The lanthanide and phosphate solutions were adjusted to pH ∼2 prior to the synthesis using hydrochloric acid or deuterated hydrochloric acid (DCl). In the case of D_2_O solutions, the hydrate water molecules of the lanthanide salts contribute about 0.03% H_2_O by volume to the solvent. The nanocrystals were characterized using TEM imaging (Philips Tecnai F20, 200 kV).

### Preparation of Seed NCs

Small, roughly spherical ∼10 nm NC seeds were produced by taking 1 mL of a 2:1 precursor solution as described above and adding 1 mL of acetone at room temperature, which reduces the precursors solubility and cause instantaneous formation of the seed particles. This was followed by centrifugation at 1070 RCF for 5 min to separate the seed NCs and re-dispersion in 1 mL of D_2_O. Assuming high yield in the seed NC formation process, we estimate that a 100 μL seed solution would contain the equivalent of 0.1% lanthanide precursor ions. Since the seed particles are much smaller than the fully grown NCs, the number density of the seed particles in this volume is roughly of the same order of magnitude as the NC number density obtained in unseeded syntheses.

### In Situ Luminescence Measurements

A home-built luminescence measurement setup was used to measure the luminescence of Eu^3+^-doped TbPO_4_·H_2_O NCs as they are being formed, under temperature-controlled conditions. The cylindrical synthesis vial was kept at the desired temperature using a temperature-controlled aluminum block with holes for the excitation beam and emission collection, and the solution was magnetically stirred during the measurements to ensure concentration uniformity. The samples were excited using a focused high-power near-UV LED light source (Hamamatsu, 365 nm, 1.4 W/cm^2^). The emitted light was collected at 90° with respect to the excitation axis, passed through a monochromator, and detected by a photomultiplier tube. Most of these experiments were done with a 2:1 phosphate:lanthanide ion ratio.

### ^31^P NMR Measurements

The synthesis vial was taken out from the temperature-controlled aluminum block at desired sampling times and immediately quenched by dipping the vial in an ice water bath and then maintained in a refrigerator at 4 °C until NMR measurements. For better phosphate binding sensitivity, the syntheses for the NMR experiments were mostly done with a 1:1 phosphate:lanthanide ratio. The NMR measurements were performed on an AVANCE III HD Bruker spectrometer operating at a magnetic field of 11.7 T, using a 5 mm BBFO probe. The chemical shift scale is relative to that of pure H_3_PO_4_. All the samples were measured with the lock signal manually set to C_6_D_6_ (we added a capillary with C_6_D_6_ to each sample) to avoid the effect of PH or temperature on the ^31^P signal.

## Results and Discussion

### Photoluminescence Kinetics

The fully grown NCs were rod-shaped single crystals with lengths of the order of 500 nm and widths in the range of 10–50 nm (see Supporting Information, Figure S1).

The UV light (365 nm) excited the Tb^3+^ ions. In the formed NCs, the Tb^3+^ ions transfer the excitation energy to the dopant Eu^3+^ ions, which subsequently emit at various ^5^D_0_→^7^F_J_ (*J* = 0–6) transitions,^16^ with a characteristic lifetime in the order of milliseconds.^17,18^ The Eu ions have negligible absorption at the excitation wavelength and thus cannot emit unless excited by energy transferred from excited Tb^3+^ ions. Hence, this energy transfer only occurs in formed NCs where Eu and Tb ions are locked in proximity and not in the solvated Eu^3+^ ions (see Supporting Information, Figure S2). A similar idea was previously used to study the formation of Eu-doped YVO4 NCs.^19,20^

This combination of excitation, energy transfer, and emission enables us to differentiate the forming NCs from unreacted precursors. Therefore, the intensity of the Eu^3+^ emission signal is thought to be proportional to the amount of Eu^3+^ ions incorporated in the NCs, which depends on both the concentration and average size of NCs, assuming that the distribution of Eu^3+^ is uniform throughout the NCs. However, it should be noted that when prepared in a H_2_O environment, it is expected that in very small Eu^3+^-doped TbPO_4_·H_2_O NCs, where most Eu^3+^ is near the surface, there will be some quenching of its luminescence by water molecules’ vibrational overtones.

**Figure 1:**
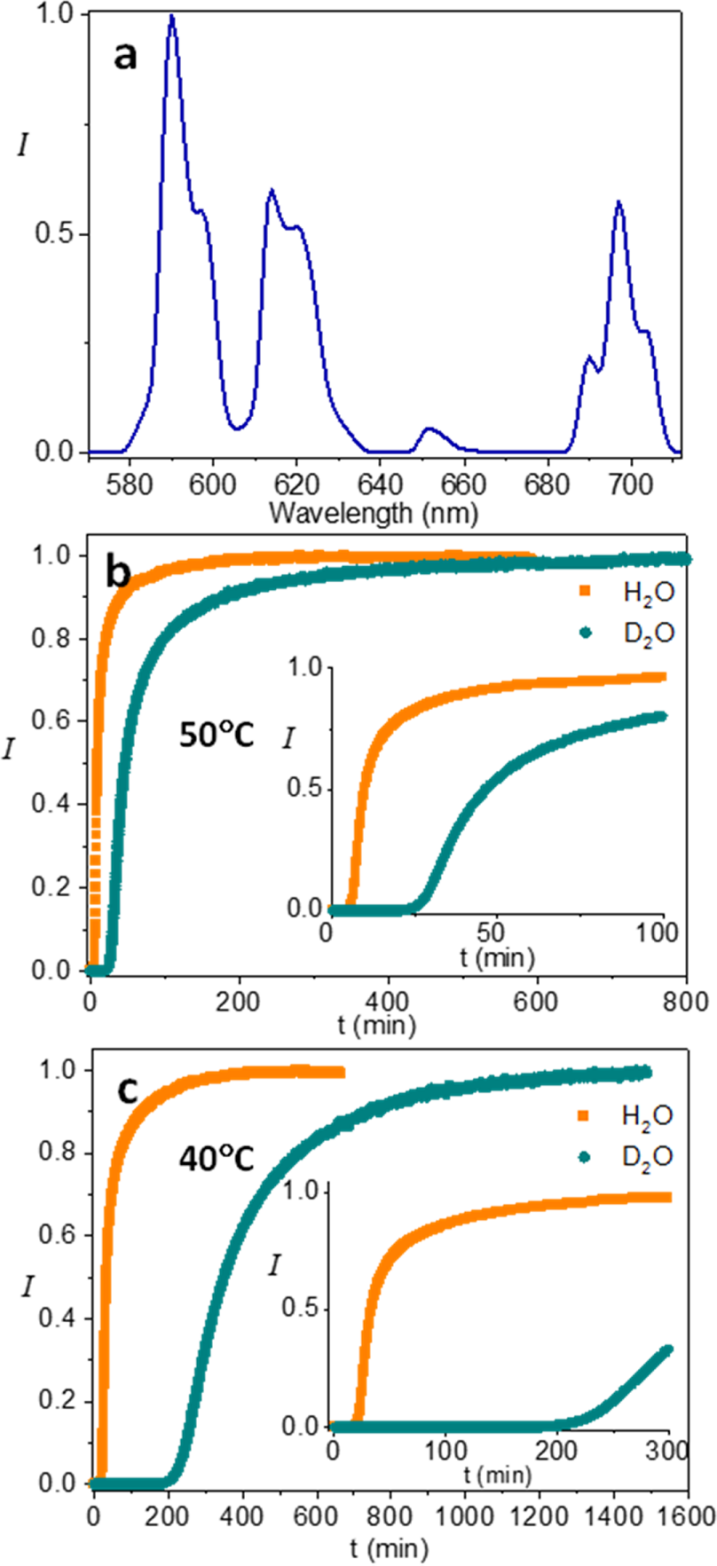
(a) Luminescence spectrum (excitation wavelength = 365 nm) of a colloidal suspension of Eu^3+^-doped TbPO_4_·D_2_O NCs with 2:1 PO_4_^3–^:Ln^3+^ precursor ratio. Emission intensity was normalized to the strongest peak. (b) Luminescence of Eu^3+^ in D_2_O and H_2_O-based NC synthesis solutions over time at 50 °C, measured at 704 nm. The 704 nm emission line is attributed to the ^5^D_0_ → ^7^F_4_ transitions of the Eu^3+^ ion. (c) Luminescence of Eu^3+^ in D_2_O and H_2_O-based NC synthesis solutions over time at 40 °C, measured at the same wavelength. The insets of panels (b) and (c) show the expanded initial stages of the reaction. All other reaction conditions were identical, and the final concentrations of the NCs were similar, as verified by their luminescence intensities. The curves in panels (b) and (c) were normalized to the emission intensity at the end of the measurement.

A Eu^3+^ luminescence spectrum of the NCs is shown in Figure 1a. The emission vs time curves (704 nm line, ^5^D_0_ → ^7^F_4_ transition) comparing NC formation kinetics in H_2_O and D_2_O at two temperatures (50 and 40 °C) are shown in Figure 1b,c. An induction period, where no Eu^3+^ luminescence occurs, was observed followed by a steep increase reflecting a fast NC nucleation and growth phase, up to (near) saturation. It can be seen that the curves for the two isotopes significantly differ both in the length of their induction period as well as the NC growth rate. Longer induction times and slower growth rates were obtained for D_2_O solutions. When the temperature was increased from 40 to 50 °C (Figure 1b,c), both induction times shortened. It should be stressed that since the Eu^3+^ luminescence quenching is negligible in the D_2_O solutions and considering that in the H_2_O-based process, the NC growth starts at shorter times, it can be assumed that the initial induction period in both samples does reflect a delay in the nucleation of the NCs (rather than NC emission quenching). This delay should probably correspond to some slow transformation of reactant(s) into other species, which are the precursors for the formation of the NCs, crossing a critical precursor concentration toward the end of the induction period. The kinetics of the NC formation in D_2_O and H_2_O solutions were also measured at 60 °C (see Supporting Information, Figure S3), but at higher temperatures, it was too fast to quantitatively determine the magnitude of KIE due to fast NC growth in H_2_O.

To understand the observed KIE we should consider the acidity of the NC growth medium (pH ∼2). At this pH, the phosphate ions are expected to be mostly fully protonated, in the form of H_3_PO_4_, as the first p*K*_a_ of phosphoric acid is 2.15.^21^ Hence, there is a competition between binding the Tb^3+^ (or Eu^3+^, which is chemically equivalent) and the protons attached to the phosphate ions in order to form the terbium phosphate crystal. This leads to the proposition that a major part of the KIE should be related to the strength of binding of protons vs deuterons to the phosphate ions. Other, perhaps more moderate contributions to the KIE could be related to differences in solvation energies of the lanthanide and phosphate ions in the two solvents. It is well-known that generally, deuterium binds more strongly than hydrogen due to the lower zero-point vibrational energy of the D–R bond relative to H–R.^22^ Such a difference would form a larger energy barrier for attaching a phosphate ion to Tb^3+^ from D_3_PO_4_, compared with H_3_PO_4_, and consequently slow down the formation rate of TbPO_4_ in a D_3_PO_4_/D_2_O environment.

**Figure 2:**
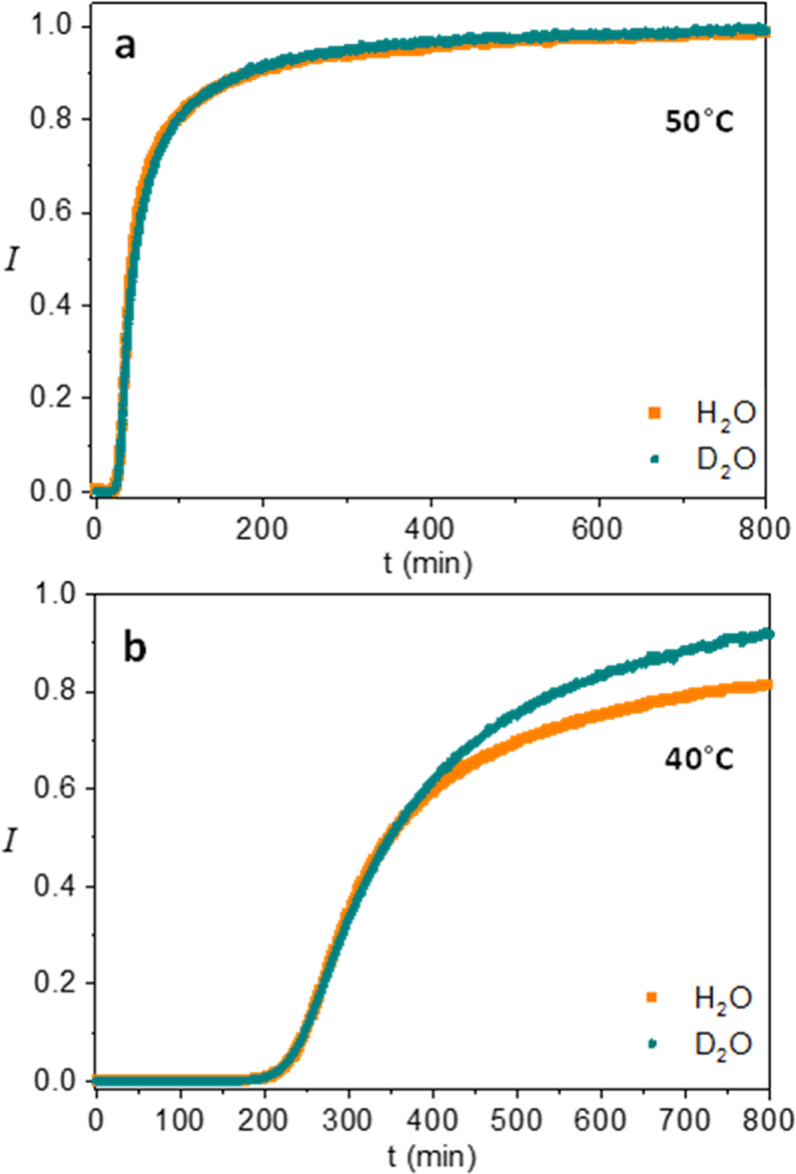
(a,b) Scaling behavior of the normalized emission intensity of Eu^3+^ of D_2_O and H_2_O solutions with a 2:1 PO_4_^3–^:Ln^3+^ precursor ratio, at 50 and 40 °C, where the H_2_O time axis is multiplied by factors of 4.5 and 10.5 for the curves taken at 50 and 40 °C, respectively.

Figure 2 shows the same curves as in Figure 1, after multiplying the time scale of the fast (H_2_O) process by the ratio of the D_2_O/H_2_O induction periods, as determined by the onset of luminescence. This scaling brings the H_2_O curve to roughly overlap the D_2_O curve, especially along the early part of NC formation. This scaling behavior of the two kinetic curves of D_2_O and H_2_O solutions both at 50 and 40 °C indicates that a similar KIE operates in the induction, nucleation, and NC growth phases. It also lends further support to the above conclusion that the quenching of Eu^3+^ emission at early growth stages in the H_2_O case is not significant. Otherwise, the scaling of the H_2_O and D_2_O curves should not be possible (see also Supporting Information, Figure S4).

**Figure 3:**
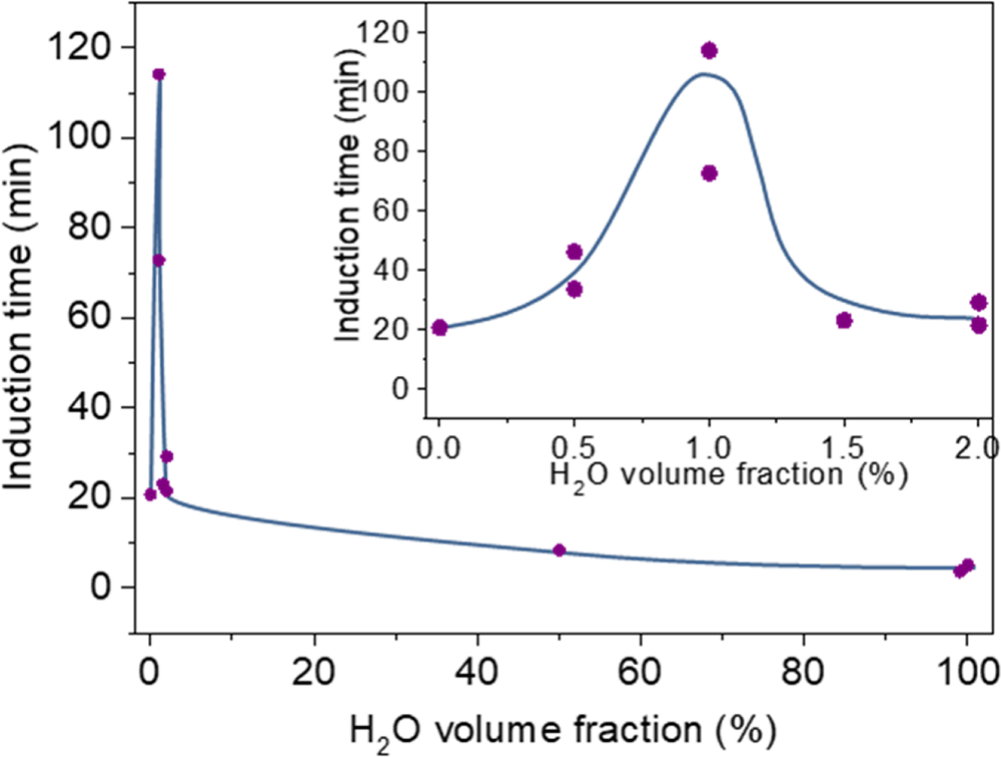
Induction period as a function of volume fraction of H_2_O in D_2_O solution with a 2:1 PO_4_^3–^:Ln^3+^ precursor ratio at 50 °C. The inset shows the lower water concentration regime expanded. The lines are hand-drawn as guides for the eye. Induction times were measured twice for some of the concentrations, to provide an estimate of the uncertainty level, which seems to be roughly of the order of ∼30% of the induction time value.

In addition to measurements in the two pure H_2_O or D_2_O solutions (where the pure D_2_O contains ∼0.03% residual H_2_O), we conducted the kinetic studies in binary mixtures of D_2_O and H_2_O with different volume ratios at 50 °C. Interestingly, while in the 50:50% case, the induction period was in between the induction periods of the pure solvents (see Figure 3), at low H_2_O concentrations (<2%), the induction times were longer than the induction period of nearly pure D_2_O. Figure 3 displays the induction time vs the H_2_O volume % (in D_2_O). A large increase in the induction time can be seen, sharply peaking at a water concentration of ∼1%. No such effect was found near 100% H_2_O. 1% D_2_O in H_2_O had an induction period very close to that of 100% H_2_O. This non-monotonous, highly H_2_O concentration-dependent behavior of the NC formation kinetics as a function of H/D ratio might indicate that the KIE is influenced by more than a single isotope effect (such as the barrier for release of protons from phosphate ions) in the NC formation mechanism.

### NMR Experiments

**Figure 4:**
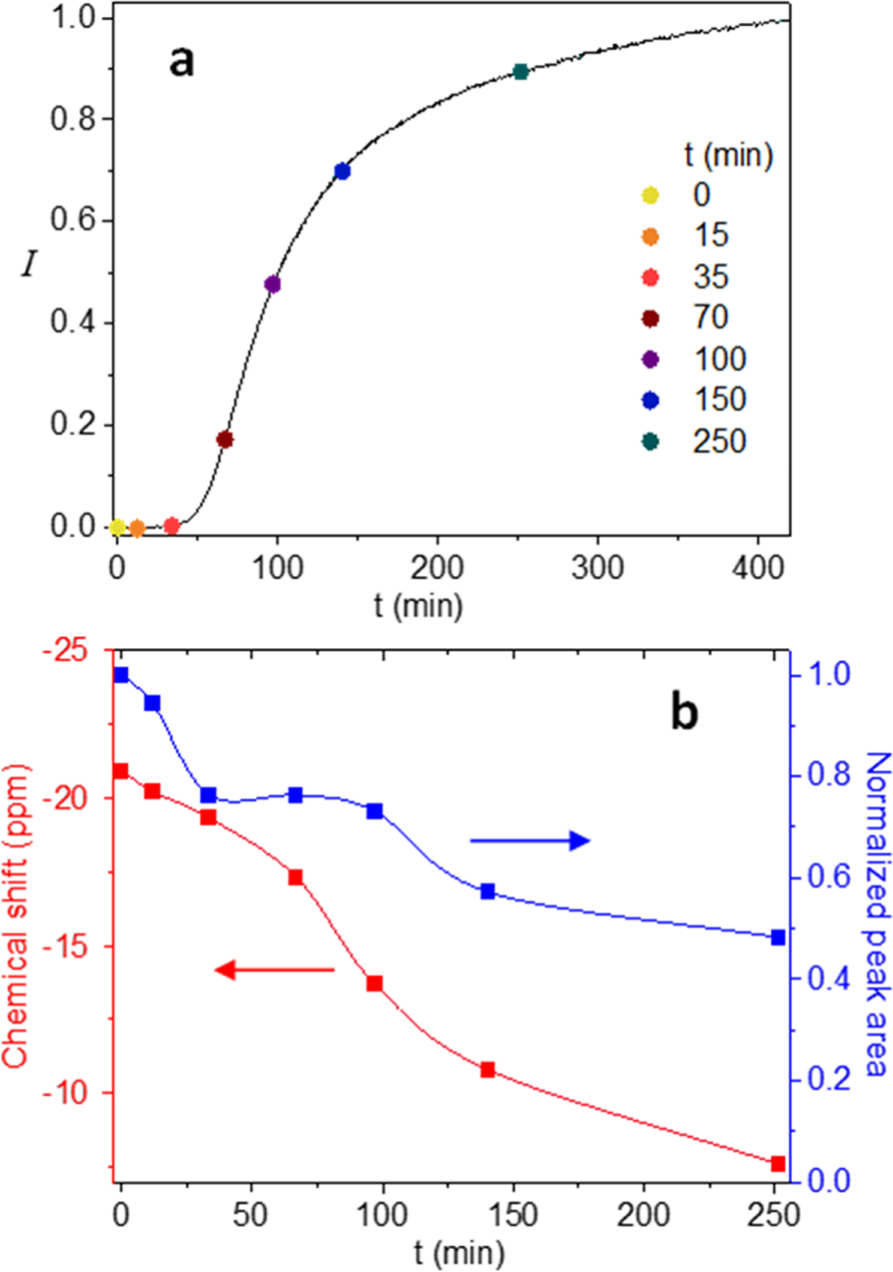
^31^P NMR results for the NC growth process with a 1:1 PO_4_^3–^:Ln^3+^ precursor ratio performed in D_2_O solution at 50 °C. (a) Eu^3+^ luminescence vs time curve with points indicating sampling times for the NMR experiments. (b) ^31^P peak chemical shift and peak area vs time for the experiment shown in panel (a). The peak integral was normalized to the peak area at *t* = 0.

In order to obtain more information on the nature of the induction period and perhaps on the NC growth period, we performed ^31^P NMR measurements of the D_2_O solutions sampled along the NC growth curves. For this purpose, NC formation experiments were run at 50 °C and quenched by cooling in an ice-water bath at different time intervals from the start. Since the 2:1 PO_4_^3–^:Ln^3+^ precursor ratio used in the KIE luminescence studies had a large unbound phosphate excess, thus reducing the chemical shift and peak area sensitivity to the amount of bound phosphate, we have focused on NMR studies with a 1:1 ratio (see Supporting Information, Figure S5). Figure 4 displays the ^31^P NMR results for the 1:1 PO_4_^3–^:Ln^3+^ case, where Figure 4a shows the NC luminescence growth curve in this case, displaying an induction period of ∼35 min in D_2_O solution, roughly 1.5 times longer than the case of the 2:1 precursor ratio (the growth kinetics is roughly first order in both ions). This was also the case for the H_2_O solution (see Supporting Information, Figure S6a). We also observed a scaling behavior of the two kinetic curves of D_2_O and H_2_O solutions, showing a similar behavior to the 2:1 PO_4_^3–^:Ln^3+^ precursor ratio.

In Figure 4b, one can see the time evolution of the phosphorus chemical shift and normalized peak area at the sampled intervals marked on the curve of Figure 4a. The peak area, normalized to the *t* = 0 value, shows that as the NCs are formed, part of the phosphorus is disappearing from the NMR spectrum (up to ∼50% at 250 min, when most of the reaction seems to have been completed). We assume that the disappearing phosphorus has been embedded in extended clusters/NCs, where its rotational correlation time becomes long, consequently reducing the transverse relaxation time until it becomes too broad to observe. Interestingly, about half of the decline in the phosphorus signal (almost 25%) occurs during the induction period, which indicates that almost half of the phosphate in the NCs emanated from pre-nucleation clusters.

In addition, the chemical shift of the phosphorus seems to be continuously declining (in absolute value) toward 0 ppm (the chemical shift of pure D_3_PO_4_) as the reaction progresses, also through the induction period. This decline is due to the decreasing concentration of free lanthanide ions in solution as they become bound to clusters/NCs. The lanthanide ions are the source of the negative shift due to their strong paramagnetic nature, and the NMR shifts are an average value that results from fast exchange between bound and unbound phosphates.^23^ The relatively slow decline in chemical shift during the induction period, relative to the decline in peak area, is probably due to the relatively fast exchange of phosphate ions in the pre-nucleation clusters/polymers, as probably the majority of bound phosphate (and lanthanide ions, see Supporting Information, Figure S4) is in contact with the solution. When the NCs start to form (∼35–100 min), the peak area remains roughly constant, while chemical shift reduction accelerates. We attribute the latter effect to the reduced exchange rate as the phosphate is being trapped in the growing NCs. The reason for the plateau in peak area vs time at this regime might be related to particle attachment as the main growth mechanism at this stage. In such a case, the number of bound phosphate ions would not change significantly, as the growth mostly consumes pre-formed clusters or small NCs. Note that a similar trend of peak area vs chemical shift was observed in the 2:1 Tb:phosphate precursor ratio experiment (Supporting Information, Figure S8); hence, it is a reliable mechanistic indicator. Beyond 100 min, the gradual growth appears to be consuming free precursor ions, as both the peak area and chemical shift reduce concomitantly.

It seems that within the time window of the NMR/photoemission measurements, only about half of the precursors react to form the NCs, and then the reaction seems to slow down, with an additional slow increase of luminescence beyond the measurement time. We believe that the growth might be slowing down along the NC formation due to slight acidification of the solution because of release of protons/deuterons from the H_3_PO_4_ or D_3_PO_4_ as PO_4_^3–^ ions are being incorporated in the growing NCs. Also in the case of 2:1 PO_4_^3–^:Ln^3+^, only about ∼50% of the Ln^3+^ ions (25% of the PO_4_^3–^ ions, see Supporting Information, Figures S7 and S8) seem to have reacted within the measurement time.

The spin relaxation time *T*_1_ does not change significantly during the induction period, while during the NC growth, *T*_1_ becomes longer due to reduction in free lanthanide ion concentration (see Supporting Information, Tables S1 and S2). This is another indication that during the induction period, when in the pre-nucleation cluster/polymer form, the phosphate ions still experience a fast exchange rate between bound and unbound phosphate in the solution, and Tb^3+^ ions in the clusters may still be in exchange, although at a slower rate. When the clusters are converted into NCs, most of the Tb is unable to be exchanged any longer.

The findings of the NMR experiments indicate that the induction period may be characterized by the growth of nonluminescent, disordered clusters or polymeric structures involving both phosphate and lanthanide ions. In those initial (dynamically) disordered clusters, the europium ions do not spend enough time in proximity to the terbium ions to allow for efficient energy transfer and thus no Eu^3+^ luminescence is observed.

### Seeded Growth of the NCs

**Figure 5:**
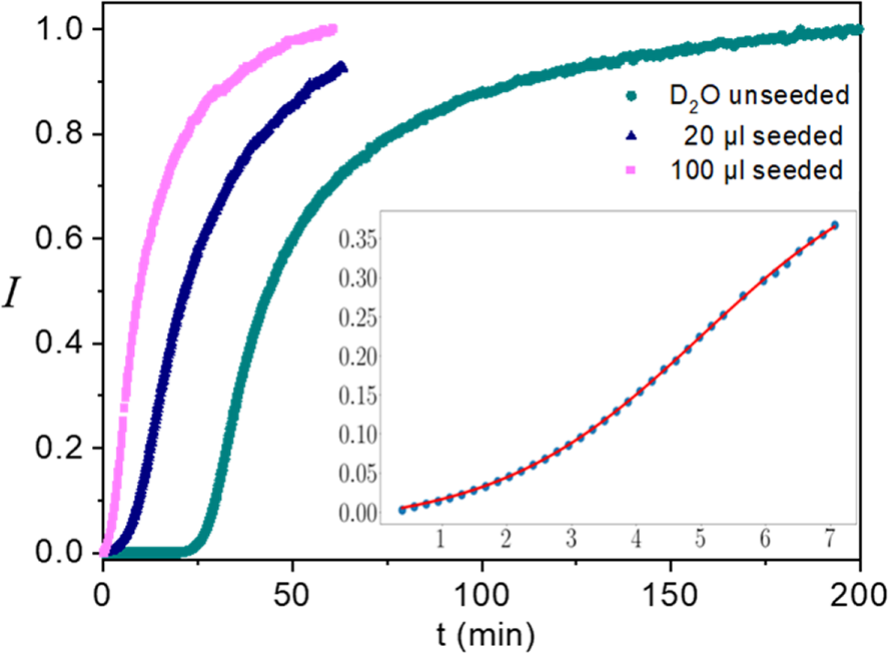
Luminescence vs time measured at 50 °C in D_2_O, following addition of two different quantities of NC seed particles (20 and 100 μL of seed solution), compared with an unseeded process. Inset: expanded view of the first 7 min of the 100 μL seeded growth process (dots) together with the fitted Finke–Watzky model (red line).

In order to better separate between the induction and NC growth processes, we have performed another set of luminescence vs time kinetic measurements, where we introduced to the precursor solution small (∼10 nm) pre-formed TbPO_4_·H_2_O NCs. Figure 5 displays the comparison of the growth kinetics in D_2_O with and without the seed particles, showing the absence of an induction period in the seeded cases. However, it can be seen in the inset of Figure 5 (and in Supporting Information, Figure S9) that the luminescence increase undergoes an inflection point and thus seems to be still governed at early growth stages by two processes. This time, as shown in Figure 5 (and Supporting Information, Figure S9), the early parts of the curves measured both at 40 and 50 °C could be fitted by the Finke–Watzky model equations, yielding two rate constants. The first constant, *k*_1_, governing the initial growth kinetics is probably related to formation of initial precursor clusters (see values in the Supporting Information, Table S3). The second rate constant, *k*_2_, reflects the fast growth mechanism, which seems to occur by cluster/NC attachment.

### Discussion of NC Formation Mechanism

Consequently, it seems that the NC formation mechanism in the present case should be discussed in the context of pre-nucleation clusters models,^24,25^ as often observed for crystallization in various molecular systems,^26^ and more specifically, for calcium carbonate crystal nucleation.^27,28^ Since the strong KIE seems to occur also during the fast NC growth phase, which follows the induction period (scaling behavior shown in Figure 2), we believe that the transition from the disordered clusters to crystal nuclei should probably also involve detachment of some protons bound to phosphate ions (as is probably the case in the NC growth phase).

It seems that neither of the two models (La Mer or Finke–Watzky) could purely describe the unseeded NC growth, as the sigmoid-like growth curve could not be satisfactorily fitted by Finke–Watzky model’s expressions, even when shifted by the induction period. In an attempt to roughly estimate kinetic energy barriers for the two isotopes, we inserted the ratios of induction times into the Arrhenius equation for pairs of temperatures (Supporting Information, Table S4). The activation energies that were obtained were 122 ± 25 kJ/mol for H_2_O and 150 ± 30 kJ/mol for D_2_O, but the relatively large uncertainty in these values makes this estimate fairly qualitative.

In the seeded growth case, fitting of the early growth stages in D_2_O with Finke–Watzky’s model equations was successful and a similar activation energy value was obtained for the presumed initial clustering process (143 ± 10 kJ/mol, see Supporting Information, Table S3 and Figure S9). The extracted activation energy for the NC growth process was 205 ± 10 kJ/mol.

As mentioned above, the KIE could be related to differences in the binding energies of the proton/deuteron to the phosphate ions. The vibrational frequency of the PO–H bond is roughly ∼3660 and ∼2700 cm^–1^ for the PO–D bond.^29^ The difference of the zero-point vibrational energy of PO–D bond relative to PO–H is half of the vibrational energy quanta difference, i.e., ∼6 kJ/mol (∼500 cm^–1^). The scale of difference in activation energy between D_2_O and H_2_O that we obtained was of the order of 30 kJ/mol, i.e., a bit larger than the energy difference required for breaking 3 deuterons minus 3 protons from the phosphate (i.e., H_3_PO_4_ → 3H^+^+PO_4_^–3^), assuming that most of the activation energy difference comes from the zero point energy difference. Other contributions to the isotopic difference in activation energy could be related to ion solvation energies.

Consequently, the information provided by the luminescence and NMR vs time studies of the KIE indicates that the La Mer type model would not be suitable for the present case, as nucleation does not seem to be a simple case of supersaturation. Assuming the formation of pre-nucleation polymers/clusters consisting of phosphate and lanthanide ions during the induction period, it becomes clear that the nucleation step is some sort of a phase transformation, which occurs when some critical size of the polymer/cluster is achieved. The Finke–Watzky model has been used in the past to describe phase transformations;^30^ hence, it might be possible to modify it to properly describe the current NC nucleation and growth process (for the unseeded growth case).^31^ The scaling behavior of the growth curves for both isotopes shows that both induction and growth processes experience similar KIE; hence, they might also be similar in their chemical nature. It is conceivable that in both cases, loss of protons or deuterons is the major kinetic energy barrier for precursor aggregation, where in the induction case, it is aggregation into polymer/cluster and in the NC growth, it is simply addition of building blocks to the NC.

Both the NMR data and seeded growth experiments point to a particle attachment mode of NC growth at early growth stages. It is yet to be determined which type of particle aggregation occurs.^32^ In the case of first transforming to small NCs, the mechanism must occur via oriented attachment,^33^ as the formed NCs are single crystals. However, on the basis of the strong symmetry breaking previously observed in the growth of these NCs,^15^ we can conclude that the oriented attachment of small NCs is much less likely to occur, as such a process would not lead to the observed autocatalytic-type chiral amplification. Hence, the most likely growth mechanism would be the attachment of the disordered clusters/polymers to the NCs followed by their transformation to crystalline as they unify with the core. The activation barrier for the growth by the cluster attachment process seems to be higher than the cluster formation barrier, perhaps also involving the loss of a number of surface protons/deuterons as well as some structural rearrangement processes.

The significant slowing down of the reaction rates at low H_2_O concentration in D_2_O is a unique feature, which is currently of unknown origin. Further studies of this effect, using molecular dynamics simulations including quantum effects for the protons/deuterons, for example, would be valuable for better understanding of the KIE and consequently also of the NC formation mechanism.

It should be noted that all the phenomena described in this work are relevant for the NC growth occurring around pH ∼2. At pH values higher than 3, the NC growth speeds up significantly and produces very long, narrower, defective TbPO_4_·H_2_O nanowires.

## Conclusions

We have demonstrated an acidic NC growth process exhibiting a strong H/D KIE. Studying the NC growth kinetics by photoluminescence and NMR enabled us to conclude on the formation of pre-nucleation clusters or polymers consisting of the phosphate and lanthanide ions and on initial NC growth stages involving particle attachment. The scaling behavior of the growth curves in H_2_O and D_2_O hints at similar KIE effects occurring both at the induction and NC growth phases, probably involving proton/deuteron detachment from the phosphate ions.

This study was enabled by the unique physical properties of the paramagnetic lanthanide ions, which are excellent luminescent reporters of their environment, and their localized, robust emission properties enable quantitative assessment of the NC formation process. We believe that this type of NCs may serve as an excellent model system for further studying and gaining a deeper understanding of crystal nucleation and growth processes.

Considering that the formed Eu^3+^-doped TbPO_4_·H_2_O NCs are also chiral and may be either left- or right-handed, continuing this study to follow the enantiomer evolution with time along the NC formation curves could further teach us about the NC formation mechanism and in particular symmetry breaking and large chiral amplification previously reported for this system.^15^

## Supporting Information Available

Additional luminescence spectra and kinetic curves, raw NMR data and analysis, and calculation of activation energies (PDF)
