# Supplementary material for: A Kinetic Isotope Effect in the Formation of Lanthanide Phosphate Nanocrystals
Source: J Am Chem Soc. 2022 May 20;144(21):9451–7. doi: 10.1021/jacs.2c02424 (PMC9189826; doi:10.1021/jacs.2c02424)
Supplement: Supplementary file 1 — ja2c02424_si_001.pdf [file ja2c02424_si_001.pdf]

Supporting Information for

A kinetic isotope effect in the formation of lanthanide phosphate nanocrystals

Gal Schwartz,[1] Uri Hananel,[1] Liat Avram,[2] Amir Goldbourt,[1] Gil Markovich[1]*

[1] School of Chemistry, Raymond and Beverly Sackler Faculty of Exact Sciences, Tel Aviv University, Tel Aviv 6997801, Israel.

[2] Department of Chemical Research Support, Weizmann Institute of Science, Rehovot 7610001, Israel.

* Email address: gilmar@post.tau.ac.il

Transmission Electron Microscopy (TEM) image of the NCs

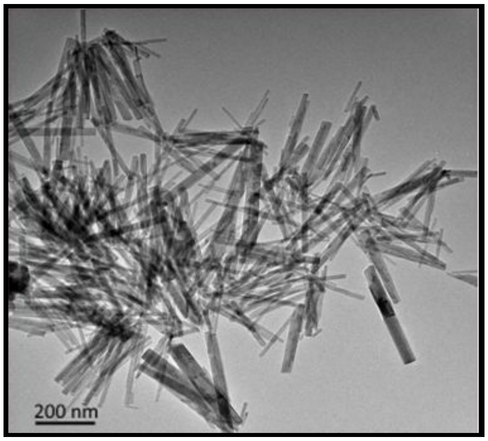

Figure S1. TEM micrograph of the synthesized Eu^{3+}-doped $TbPO_4{\cdot}D_2O$ NCs.

Luminescence spectra of the nanocrystals and their precursors

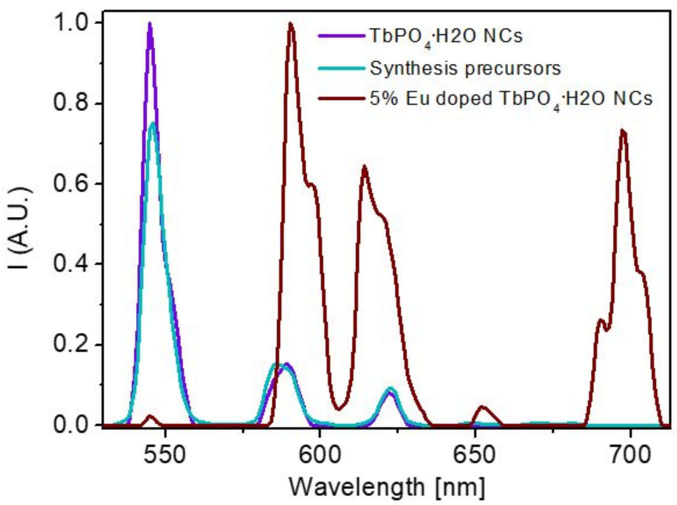

Figure S2. The luminescence spectra, $\lambda_{excitation}$= 365 nm, of undoped $TbPO_4{\cdot}H_2O$ NCs, unreacted precursors for $TbPO_4{\cdot}H_2O$ synthesis with Eu^{3+} (Tb:Eu molar ratio of 95:5%) and $TbPO_4{\cdot}H_2O$ doped with 5% Eu^{3+} NCs. As verified by their luminescence intensities, all other reaction conditions were identical and the final concentrations of the NCs were similar.

The unreacted precursors show the characteristic emission of Tb^{3+} ions in H_2O alone, due to the low Eu^{3+} concentration combined with quenching by H_2O molecules,[1,2] and with its weak absorption at the excitation wavelength of 365 nm.[3] Therefore, there is no detectable Eu^{3+} emission at our experimental wavelength (704 nm, $^5D_0 \rightarrow {}^7F_4$ transition) for the precursors solution. The 704 nm emission would only occur when energy transfer between Tb^{3+} and Eu^{3+} is enabled in formed NCs where Eu and Tb ions are in nearest-neighbor lattice positions and not dispersed in the aqueous solution.

Luminescence vs. time measurement of NC formation at 60°C

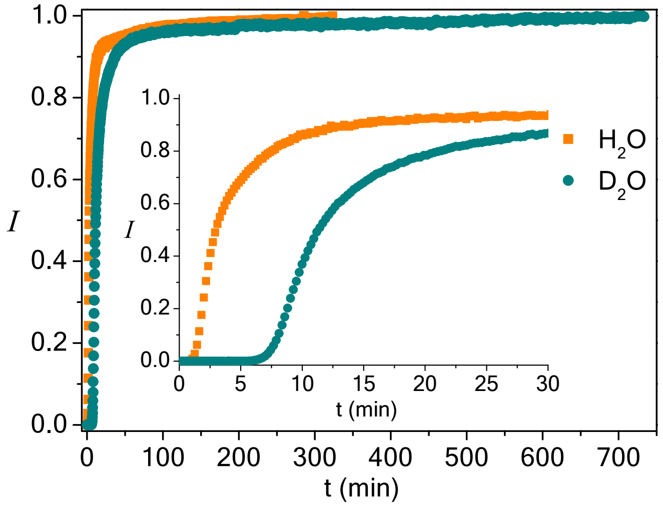

Figure S3. The luminescence spectra ($\lambda_{excitation}$= 365 nm) of Eu^{3+} for NCs grown in D_2O and H_2O solutions over time at 60°C, measured at 704 nm, $^5D_0 \rightarrow {}^7F_4$ transition. Phosphate: lanthanide precursor ratio was 2:1. The inset show the initial stages of the reaction expanded. The relative accuracy of determining the induction period for the H_2O case was low due to very small number of measured points during this period.

Fig. S3 displays typical Eu^{3+} luminescence vs. time curves, comparing NC formation in H_2O and D_2O solutions at 60°C. This was the highest temperature for which the reaction kinetics could be measured with reasonable time resolution.

Growth kinetics by direct excitation of Eu^{3+} ions

We could also follow the growth kinetics of the Eu^{3+}-doped $TbPO_4{\cdot}H_2O$ NCs by exciting the emission using a 395 nm diode laser. This provides direct excitation of the Eu^{3+} ions, without the need to transfer energy from the Tb^{3+} ions.

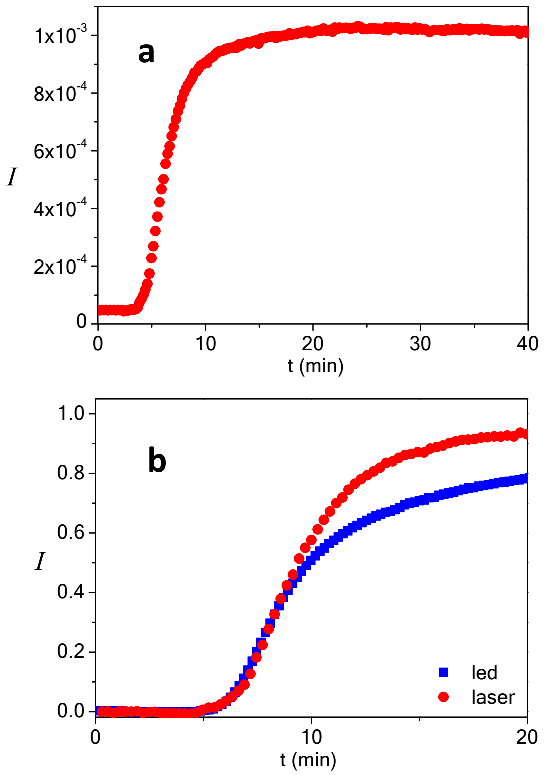

Figure S4. (a)The luminescence vs. time NC growth curve measured at 50°C in H_2O with direct europium excitation using a 395 nm (100 mW) diode laser. It can be seen that there is small and constant emission during the induction period, showing that the quenching by water molecules is not complete and that the pre-nucleation clusters/polymers have high hydration level and probably also dynamic ion exchange with the solution. (b) A comparison of the same experiment performed by direct excitation by the 395 nm laser to the one performed by 365 nm LED excitation of the Tb^{3+} ions. The direct excitation curve was shifted to zero intensity at the induction period for the comparison. The overlap of the rise of emission signal at these two experiments demonstrates the insensitivity of the results to the exact nature of excitation and probably also insensitivity to emission quenching by water at the point where the NCs start to form.

Raw NMR data for the 1:1 precursor ratio

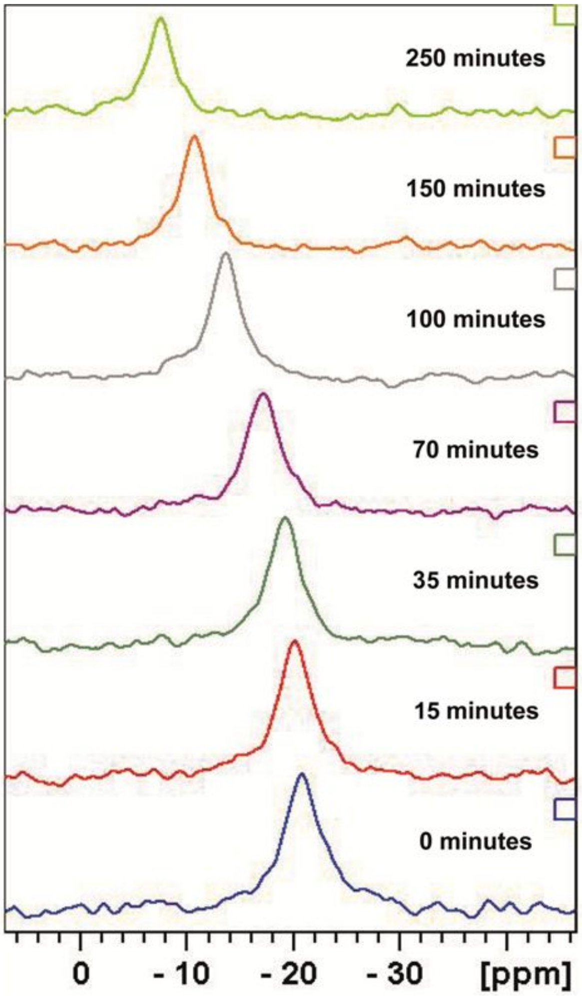

Figure S5. ^{31}P NMR (202.436MHz) signals of sampled solutions at different stages of the nanocrystal growth with 1:1 PO_4^{3-}:Ln^{3+} precursor ratio performed in D_2O solution at 50°C. All the samples were measured with the lock signal manually set to C_6D_6 (we added a capillary with C_6D_6 to each sample) to avoid the effect of pH or temperature on the shift of ^{31}P signal. Peak shifts and areas are plotted in Fig. 4 of the paper.

Luminescence vs. time measurements of NC formation with 1:1 PO_4^{3-}:Ln^{3+} precursor ratio

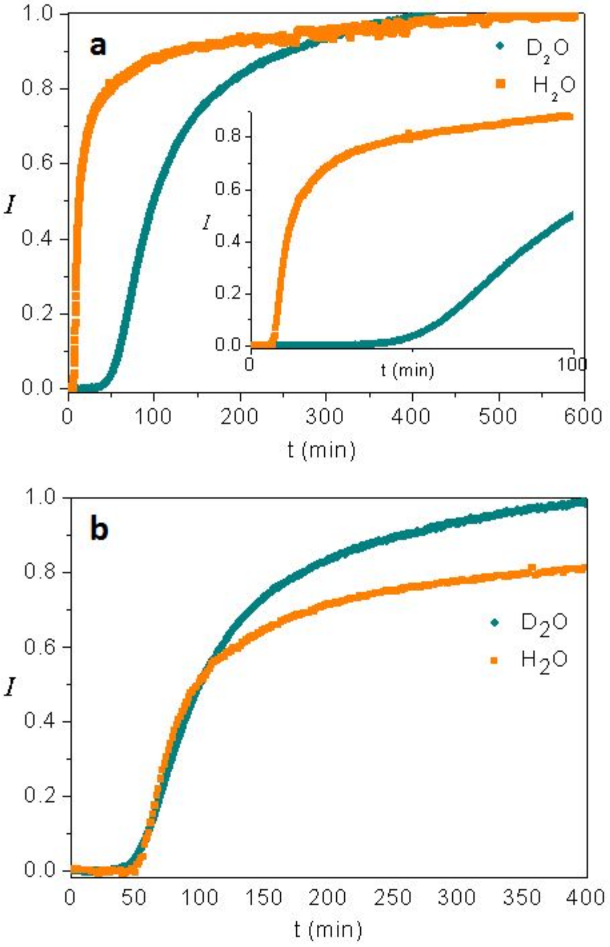

Figure S6. (a) The luminescence of Eu^{3+} vs. time for a NC synthesis with 1:1 PO_4^{3-}:Ln^{3+} precursor ratio, performed in D_2O and H_2O solutions in 50°C, measured at 704 nm ($\lambda_{excitation}$= 365 nm $^5D_0 \rightarrow {}^7F_4$ transitions). (b) The scaling behavior of the normalized emission intensity of Eu^{3+} of D_2O and H_2O solutions at 50°C at 704 nm, where the H_2O time axis is multiplied by factors of 7.4.

Figure S6 shows KIE in the formation of nanocrystals with 1:1 PO_4^{3-}:Ln^{3+} precursor ratio. In H_2O the induction period is ~7.1 minutes, which is 1.4 times longer than the case of the reaction with 2:1 precursor ratio. For D_2O, the induction period is ~34.6 minutes, which is ~1.7 higher than the 2:1 ratio. The kinetic curve shows similar stages of crystal growth, besides the last stage, which shows a slight increase of luminescence with time and not a complete saturation. The scaling behavior in Fig. S6b brings the H_2O curve to roughly overlap the D_2O curve, similar to the 2:1 precursor ratio synthesis discussed in the paper.

Raw NMR data for the 2:1 precursor ratio

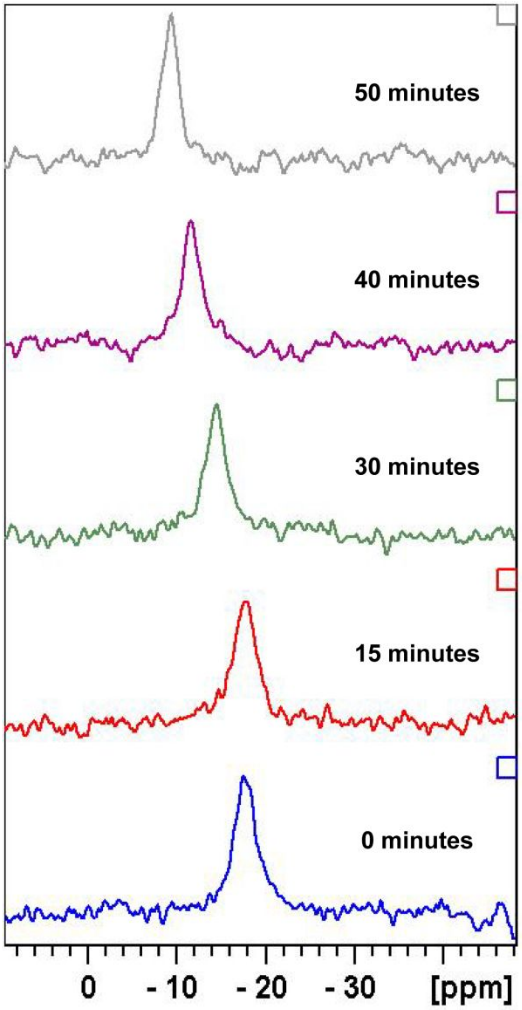

Figure S7. ^{31}P NMR (202.436MHz) spectra of sampled solutions at different stages of the nanocrystal growth with 2:1 PO_4^{3-}:Ln^{3+} precursor ratio performed in D_2O solution at 50°C. All the samples were measured with the lock signal manually set to C_6D_6 (we added a capillary with C_6D_6 to each sample) to avoid the effect of pH or temperature on the ^{31}P signal shift.

NMR results for the 2:1 precursor ratio

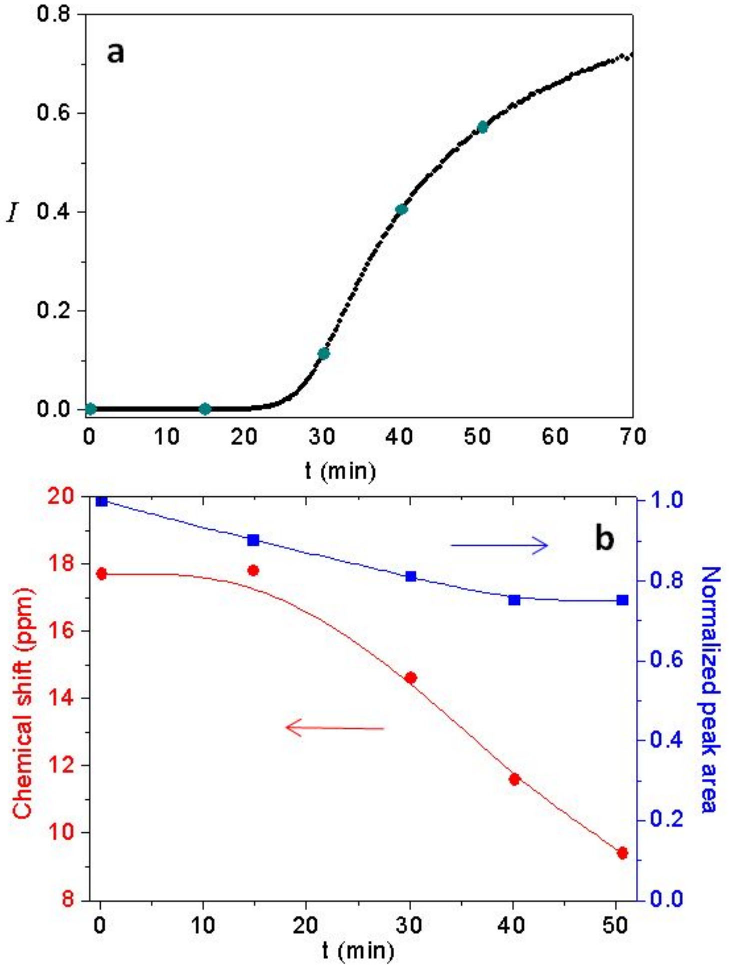

Figure S8. ^{31}P NMR results for the NC growth process with 2:1 PO_4^{3-}:Ln^{3+} precursor ratio performed in D_2O solution at 50°C. (a) The Eu^{3+} luminescence vs. time curve with points indicating sampling times for the NMR experiments. (b) ^{31}P peak chemical shift and peak integral vs. time for the experiment shown in (a).

Figure S8 shows the time evolution of the phosphorus NMR signals sampled at different stages of the NC growth with 2:1 PO_4^{3-}:Ln^{3+} precursor ratio. As observed in the case of the 1:1 PO_4^{3-}:Ln^{3+}, the peak integral decreases due to the reaction of the phosphorus within the measured time. In addition, the peak shifts towards zero. The total decrease in peak area is about 25%, corresponding to ~50% reaction yield (for the amount of lanthanide ions in solution) similar to the 1:1 case.

Spin relaxation time T_1

Sample [min]	T_1[ms]
0	21.7
15	23.1
35	24.9
70	27.7
100	29.6
150	39.6
250	54.3

Table S1. Spin relaxation time T_1 values of different sampling time with 1:1 PO_4^{3-}:Ln^{3+} precursor ratio.

Table S1 shows the increase of spin relaxation time T_1 during the induction period and the NC growth phase. At 250 min, when most of the reaction seems to be completed, T_1 is significantly longer due to the reduction in free Tb^{3+} and Eu^{3+} ions concentration.

Sample [min]	T_1[ms]
0	24.7
15	22.9
30	27.6
40	37.9
50	44.2

Table S2. Spin relaxation time T_1 values of different sampling time with 2:1 PO_4^{3-}:Ln^{3+} precursor ratio.

Table S2 shows the change of spin relaxation time T_1 as the reaction progresses. There is a general trend where the relaxation time increases with time, with the exception of the sample taken at the 15 minutes mark, during the induction period. The increase of the relaxation rates supports the reduction in the exchange between bound and unbound phosphate ions and decrease in the free lanthanide concentration with time.

Determination of activation energy for pre-nucleation cluster formation

For the induction (cluster formation) period, we assume that our rate constant is proportional to the inverse of the induction period, i.e., $k_{ind} \propto \frac{1}{t_{ind}}$. Therefore, the Arrhenius law for two temperatures can be written as:

$$(1)\ ln\frac{t_1}{t_2} = ln\frac{k_2}{k_1} = -\frac{E_a}{R}\left(\frac{1}{T_2} - \frac{1}{T_1}\right)$$

Where T is the temperature, R is the universal gas constant and E_a is the activation energy.

For measurements in H_2O, we only considered the induction time at two temperatures: 40°C and 50°C, because the reaction occurred rapidly in 60°C and extracted induction time value was unreliable. In D_2O, we considered all three temperatures (40, 50, 60°C) and took the average activation energy for the three pairs.

In the case of the seeded growth experiments performed in D_2O, we fitted the growth curves (truncated at some time after the observation of the inflection point) with the Finke-Watzky's model equation:[4]

$$(2)\ [\mathrm{B}]_{\mathrm{t}} = [A]_0\left(1 - \frac{k_1 + k_2[A]_0}{k_2[A]_0 + k_1 e^{(k_1 + k_2[A]_0)t}}\right)$$

Where $[A]_0$ is the initial precursor concentration, k_1 is the rate constant of the nucleation process (or cluster formation, in our case) and k_2 is the rate constant of the autocatalytic growth. In the generalized case, we define $k_2[A_0] = k_{eff}$, and we use the luminescence intensity so that $I \propto B$, where B is the lanthanide concentration incorporated into the NCs:

$$(3)\ [\mathrm{B}]_{\mathrm{t}} = [A]_0\left(1 - \frac{k_1 + k_{eff}}{k_{eff} + k_1 e^{(k_1 + k_{eff})t}}\right)$$

The values of rate constants k_1 and k_{eff} were extracted by fitting the Finke–Watzky two-step kinetic model to our experimental data and are given in the table S3 below:

Temperature	$k_1[min^{-1}]$	$k_{eff}[min^{-1}]$
40°C	0.005±0.003·10^{-2}	0.05±0.04·10^{-2}
50°C	0.03±0.04·10^{-2}	0.6±0.8·10^{-2}

Table S3: Rate constants obtained by fitting our data with the Finke-Watzky model at 40°C and 50°C.

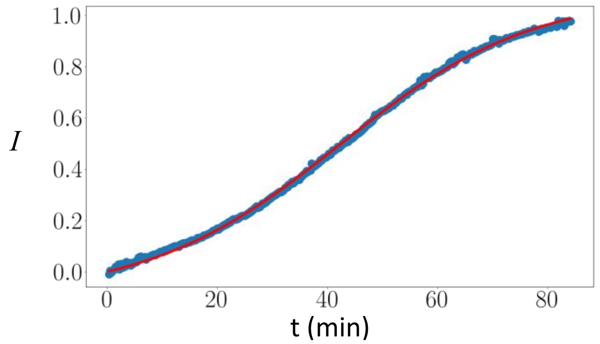

Figure S9. Emission vs. time measurement of a seeded growth experiment performed in D_2O at 40°C using 100 µL of NC seeds solution measured at 704 nm. The red line is a fit to the Finke-Watzky model. Emission intensity scale was normalized to the emission at the end of the growth process.

Then we plugged the values obtained for Finke-Watzky model's k_1 and k_{eff} at 40 and 50° in eq. 1 and got the estimate for E_a for the seeded growth in D_2O.

The activation energies for both solvents are given in the Table S4 below:

**Solvent**	**E_a [kJ/mol] for cluster formation**	**E_a [kJ/mol] for NC growth**
H_2O	122±25	-
D_2O	150±30	-
D_2O (seeded)	143±10	205±10

Table S4: Activation energies calculated from the rate constants from equation (1). The difference in activation energy between the two solvents was ~30 kJ/mol, but the uncertainty is of the same order, hence it is difficult to quantitatively conclude on the difference in activation energy between the two isotopes.

References

1. Haas, Y.; Stein, G. Pathways of radiative and radiationless transitions in europium (III) solutions: Role of solvents and anions. *J. Phys. Chem.* **1971**, *75*, 3668–3677.

2. Voloshin, A. I.; Shavaleev, N. M.; Kazakov, V. P. Water enhances quantum yield and lifetime of luminescence of europium(III) tris-β-diketonates in concentrated toluene and acetonitrile solutions. *J. Lumin.* **2001**, *93*, 191–197.

3. Binnemans, K. Interpretation of europium(III) spectra. *Coord. Chem. Rev.* **2015**, *295*, 1–45.

4. Bentea, L.; Watzky, M. A.; Finke, R. G. Sigmoidal Nucleation and Growth Curves Across Nature Fit by the Finke-Watzky Model of Slow Continuous Nucleation and Autocatalytic Growth: Explicit Formulas for the Lag and Growth Times Plus Other Key Insights. *J. Phys. Chem. C* **2017**, *121*, 5302–5312.
